# Capturing the metabolomic diversity of KRAS mutants in non-small-cell lung cancer cells

**DOI:** 10.18632/oncotarget.1958

**Published:** 2014-05-12

**Authors:** Laura Brunelli, Elisa Caiola, Mirko Marabese, Massimo Broggini, Roberta Pastorelli

**Affiliations:** ^1^ Protein and Gene Biomarkers Unit, Department of Environmental Health Sciences, IRCCS-Istituto di Ricerche Farmacologiche “Mario Negri”, Milan, Italy; ^2^ Laboratory of Molecular Pharmacology, Department of Oncology, IRCCS-Istituto di Ricerche Farmacologiche “Mario Negri”, Milan, Italy

**Keywords:** KRAS, mutations, NSCLC, metabolomics, mass-spectrometry

## Abstract

In non-small-cell lung cancer (NSCLC), one-fifth of patients have KRAS mutations, which are considered a negative predictive factor to first-line therapy. Evidence is emerging that not all KRAS mutations have the same biological activities and possible remodeling of cell metabolism by KRAS activation might complicate the scenario. An open question is whether different KRAS mutations at codon-12 affect cellular metabolism differently with possible implications for different responses to cancer treatments.

We applied an explorative mass spectrometry-based untargeted metabolomics strategy to characterize the largest possible number of metabolites that might distinguish isogenic NSCLC cells overexpressing mutated forms of KRAS at codon-12 (G12C, G12D, G12V) and the wild-type. The glutamine deprivation assay and real-time PCR were used to confirm the involvement of some of the metabolic pathways highlighted.

Cell clones indicated distinct metabolomic profiles in KRAS wild-type and mutants. Clones harboring different KRAS mutations at codon-12 also had different metabolic remodeling, such as a different redox buffering system and different glutamine-dependency not driven by the transcriptional state of enzymes involved in glutaminolysis.

These findings indicate that KRAS mutations at codon-12 are associated with different metabolomic profiles that might affect the responses to cancer treatments.

## INTRODUCTION

Lung cancer is the leading cause of cancer-related mortality worldwide [1], closely tied to smoking habits and environmental factors such as outdoor air pollution [2]. Approximately 85% of lung cancer cases are non-small-cell lung cancer (NSCLC). Most NSCLC patients have locally advanced and distant metastatic disease at presentation [3], which is associated with five-year survival rates of less than 10% and 5%, respectively. To improve clinical outcomes for patients with NSCLC, targeted therapies are increasingly being used with encouraging results, particularly in patients with specific molecular features.

KRAS is frequently mutated in largely diffused tumors, such as pancreatic, colon and NSCLC [4, 5]. These mutations lead to constitutively activated proteins locked in the GTP-bound “on” state, to the activation of the MAPK and PI3K/AKT7/mTOR pathways, and ultimately to increased proliferation and resistance to apoptosis [6].

Approximately 20-25% NSCLC patients present KRAS mutations, which are a negative predictive factor of response to first-line therapy [7]. The majority of NSCLC KRAS mutations occur at codon-12, in which the glycine (G) can be replaced by aspartic acid (D), valine (V), or cysteine (C), the G12C substitution being the most frequent. There is evidence that not all KRAS mutations are equal and the different aminoacid substitutions could confer specific biological features to the tumor. The expression of a specific KRAS mutation induces a different pattern of sensitivity to anticancer agents: in particular, the expression of G12C is associated with a reduced response to cisplatin [8].

KRAS activation supports the decoupling of glycolysis and TCA metabolism, with glutamine supplying increased carbon to drive the TCA cycle. These results provide evidence that oncogenic KRAS is involved in the metabolic reprogramming of cancer cells [9-13]. It has been reported that KRAS oncogenic substitutions in a panel of NSCLC cell lines affected protein behavior and altered the associations with downstream signaling transducers [14].

Studies of KRAS-mediated metabolism have mainly examined transformed fibroblasts [9-11] or single specific KRAS mutations in pancreatic xenografts or cells [12, 13]. They have largely focused on specific metabolite pool measurements or have relied on isotope tracing to estimate metabolic flux through a limited number of reactions. The overall metabolic deregulation driven by KRAS mutations in lung cancer and systematic characterization of the metabolic pathways active in lung cancer cells harboring different KRAS mutations are therefore still not clear.

We applied an explorative, untargeted metabolomics approach with liquid chromatography/tandem mass spectrometry (LC-MS/MS) to characterize the largest possible number of metabolites from relevant or potentially affected metabolic pathways in isogenic NSCLC cells overexpressing mutated forms of KRAS at codon-12 (G12C, G12D, G12V). This enabled us to draw metabolic portraits characterizing KRAS mutant clones in lung cancer cells. The different metabolic states associated with different KRAS mutations will help in designing more efficacy cancer therapy, aimed at exploiting metabolic differences among KRAS mutations in NSCLC.

## RESULTS

We applied unsupervised mass spectrometry-based metabolomics to discover unbiased small-molecule metabolic profiles that might distinguish human NSCLC cell line NCI-H1299 clones overexpressing KRAS mutations (G12C, G12D and G12V) from the wild-type (WT). Two independent clones were screened for each KRAS mutant. All the clones had comparable KRAS expression levels (supplemental Figure 1) and activity (data not shown).

Mass-spectral data were subjected to peak alignment and data pre-proccesing by SIEVE 1.3. Then data were analyzed for global changes, using multivariate statistics to determine group separation and to assess the number and percentage of molecular features that differed significantly in the four sample sets. OPLS-DA analysis indicated good separation of all the clones under both positive and negative ionization. Each KRAS mutant clone has its own metabolomics signature, distinct from KRAS WT. Each mutant was separated from KRAS WT, as shown by the score plots (supplemental Figure 2) supporting the evidence that independently derived mutant clones had similar metabolomic profiles related to their specific mutation. Here we report the results for one clone for each mutant, for simplicity (G12C, clone 2; G12D, clone 2; G12V, clone 9).

KRAS G12C, G12D and G12V were significantly separated from KRAS WT by respectively 74, 58 and 48 singular metabolic species, identified by database searches (METLIN, http://metlin.scripps.edu; HMBD, http://www.hmdb.ca/; LipidMaps, http://www.lipidmaps.org/) in positive and negative ionization. Supplemental Table 1 lists the significant metabolites.

**Figure 1:**
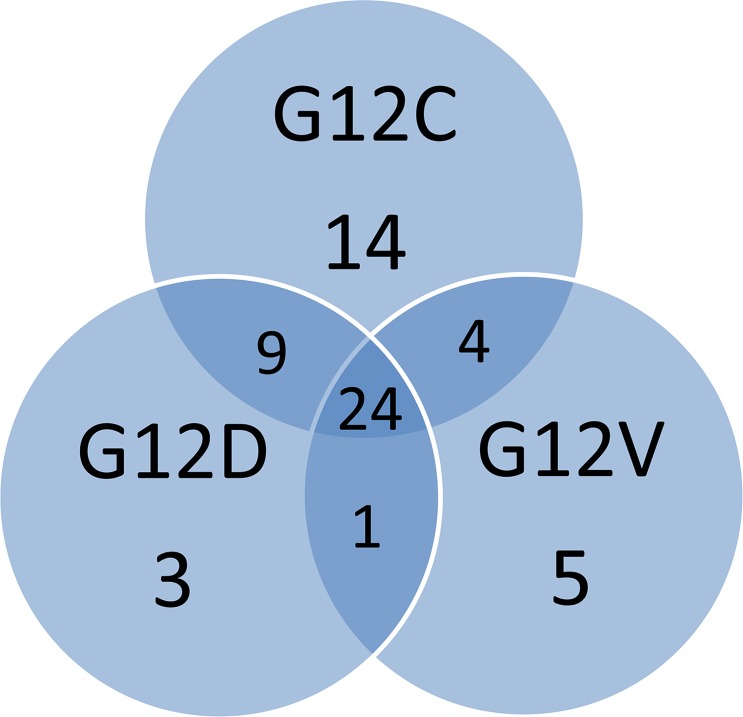
The Venn diagram shows the numbers of shared and unique metabolites identified for the overexpressing KRAS mutant clones G12C, G12D, G12V. For simplicity, figure reports the results in only one of the clones for each mutant (G12C, clone 2; G12D, clone 2; G12V, clone 9).

**Figure 2:**
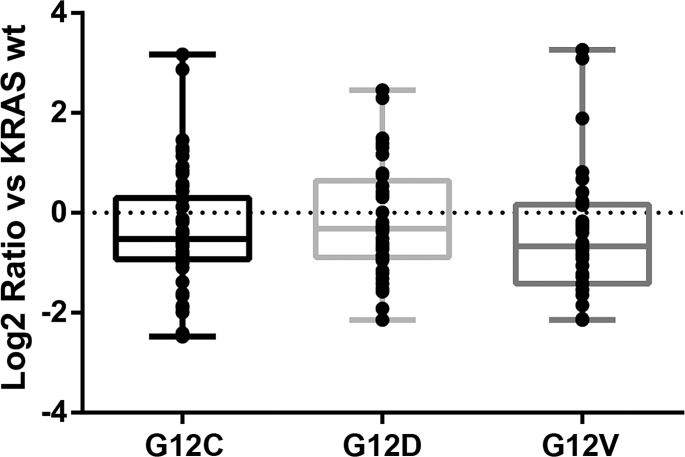
Quantitative distributions of significantly altered metabolites in overexpressing KRAS mutant clones compared to the WT counterpart. The metabolite level is expressed as the log2 ratio of relative fold change of mutant vs WT. The figure reports the results in only one of the clones for each mutants (G12C, clone 2; G12D, clone 2; G12V, clone 9).

The majority of identified metabolites were shared by all the KRAS mutant clones (Figure 1 and supplemental Table 1). Mutant G12C had the largest number of specific unique metabolites. When the relative fold changes in the significantly altered metabolites in each KRAS mutant clone were compared to the WT, it was clear that KRAS mutations generally downregulated the amount of metabolites compared to the WT clone (Figure 2), although the metabolites distribution was similar through the mutant clones.

**Figure 3:**
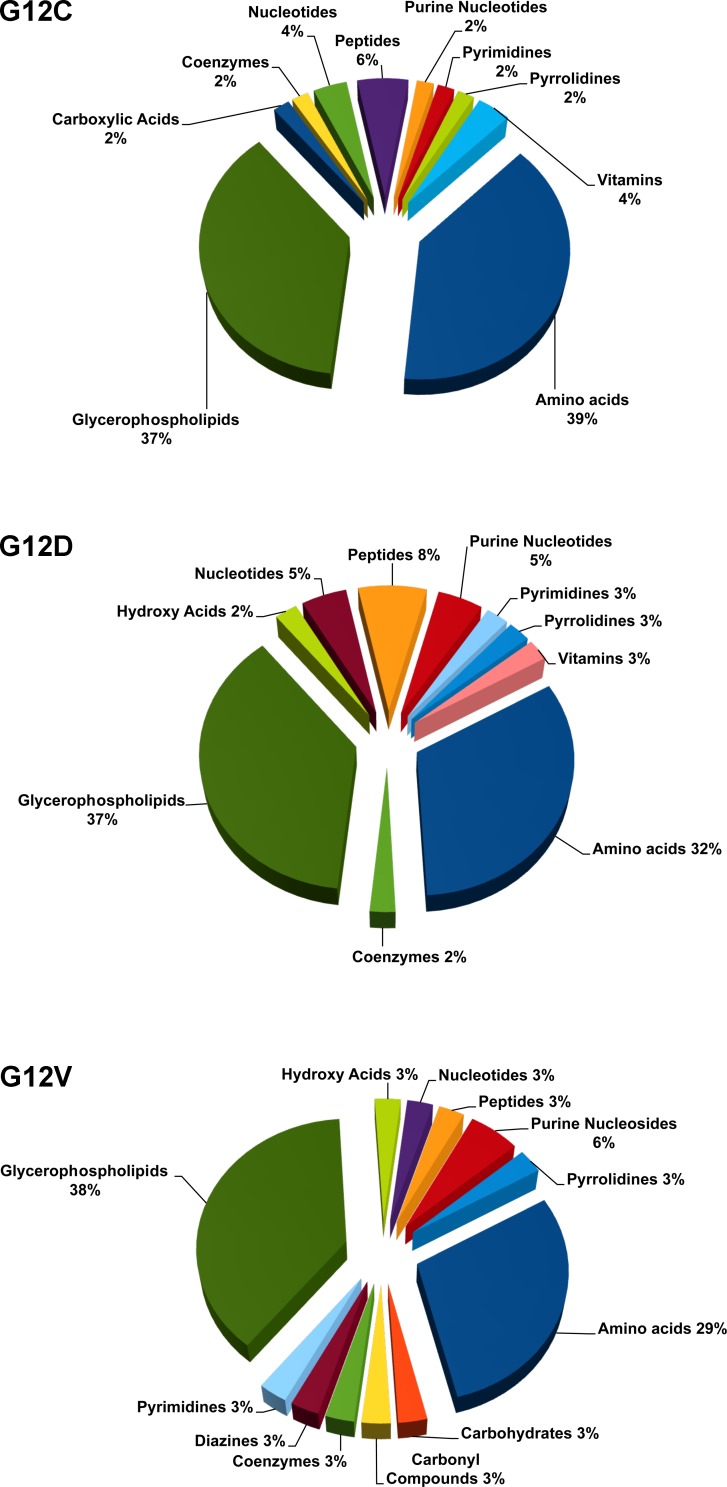
Significantly deregulated metabolites in each of the KRAS mutant clones G12C, G12D, G12V compared to WT, categorized into biochemical groupings based on the Kyoto Encyclopedia of Genes and Genome (KEGG).

To refine the comparison with the KRAS WT clone, we classified the significantly deregulated metabolites in each KRAS mutant into biochemical groups based on the Kyoto Encyclopedia of Genes and Genome (KEGG). The metabolic class distribution was similar for the different KRAS mutations, where glycerophospholipids and amino acids were the most abundant classes (Figure 3). The G12C and G12D mutations specially affected phosphatidylcholines (PC) and phosphatidylinositols (PI), whereas G12V influenced mainly PI and phosphatidylserine (PS) (supplemental Figure 3). The diversity in acyl side-chain composition and saturation was not tested with lipids standards. So we can only consider these results indicative of the distribution of major lipid classes and caution is needed in evaluating the role of these lipids.

To further interpret the biological significance of the metabolite changes in the three KRAS mutant clones, we used MetaboAnalyst tools to link metabolites to metabolic pathways. For the reasons mentioned, lipid classes were not included in the list uploaded on MetaboAnalyst.

**Figure 4:**
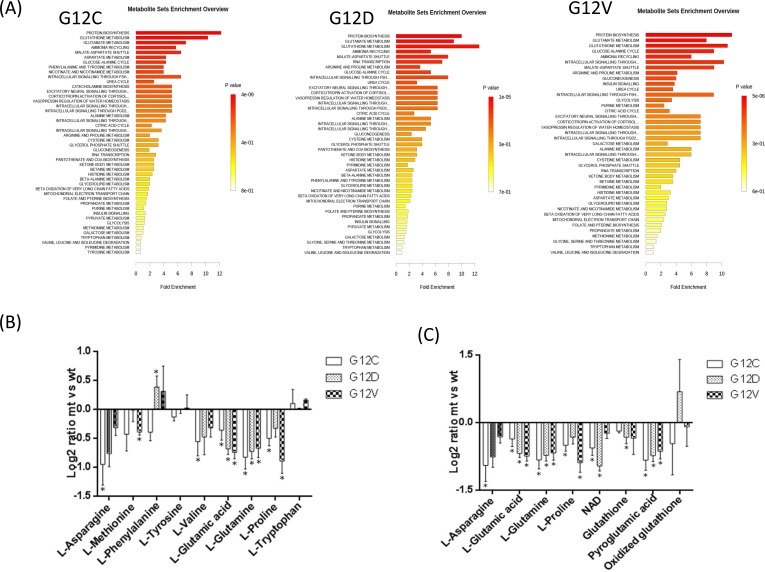
Metabolic pathway analyses related to the metabolites that specifically differ in KRAS mutant clones G12C, G12D, G12V and KRAS WT, utilizing the MetaboAnalyst functional interpretation tools. *Panel A*, graphic summary of metabolite set enrichment analysis for each KRAS mutational status. The horizontal bars summarize the main metabolite sets identified in this analysis; the bars are colored based on their *p-values* and the length is based on the -fold enrichment. *Panel B* shows the difference in abundance (as log2ratio intensity between mutant and WT) of the metabolite subset mapped into the first top-score enrichment category (protein synthesis). *Panel C* shows the difference in abundance (as log2ratio intensity between mutant and WT) of the metabolite subset mapped into three interconnected pathways categories (glutamate, glutathione metabolism, ammonia recycling). Grey bars represent the difference in metabolite abundance not significantly contributing to distinguish between KRAS mutants and WT by OPLS-DA multivariate analysis. Bars are mean SEM of the log2 ratio mt/wt of metabolite signal intensity. Asterisks mark significant differences (T-test, p<0.05) in metabolite intensities between mutant and WT clones.

**Figure 5:**
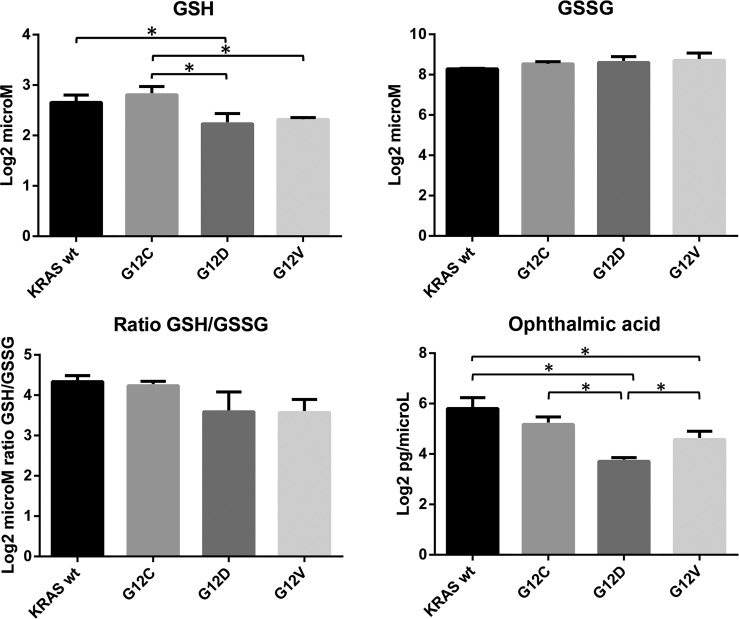
Intracellular concentrations of reduced glutathione (GSH, log2 microM), oxidized glutathione (GSSG, log2 microM), and ophthalmic acid (OPA, log2 pg/microL) in overexpressing KRAS mutant clones G12C, G12D, G12V and WT. GSH/GSSG is reported as the ratio of log2 microM GSH to microM GSSG. Asterisks mark significant concentration differences (*p<0.05, one-way ANOVA and Tukey Kramer post-hoc test). Post Test for linear trend: ratio GSH/GSSG p = 0.0023; OPA, p = 0.0018.

Figure 4 shows the metabolic pathways analyses. Panel A gives the summary plot for the metabolite set enrichment, panel B shows the difference in abundance of the metabolites mapped into the first top-score category (protein synthesis), and panel C those of metabolites mapped into the three interconnected pathway categories (glutamate, glutathione metabolism and ammonia recycling). In all three KRAS mutant clones, protein biosynthesis, glutathione, glutamate metabolism and ammonia recycling were over-represented. As shown in panel B, the level of aminoacids involved in protein synthesis was generally lower in the mutant KRAS clones than in the WT, with the exception of the greater amount of phenylalanine in the G12D clone and of tryptophan in all the KRAS mutants. Mutant clones had a smaller amount of glutamate paralleled by lower amounts of glutamine, asparagine, and proline, all metabolites interconnected in the glutamate synthase cycle. KRAS mutants also had a lower intracellular concentration of NAD^+^, an essential coenzyme regulating numerous cellular metabolic pathways (Figure 4, panel C). KRAS mutants had low levels of GSH and pyroglutamic acid, involved in glutathione metabolism. Since GSH is susceptible to oxidation during metabolite extraction and artifactual shifts may occur between GSH and GSSG, we validated their intracellular concentrations in all clones by LC-SRM-MS using a protocol that minimizes GSH loss (details in Supplemental Methods). The GSH level in KRAS G12C was slightly higher than in G12D and G12V, but not different from KRASWT (Figure 5). The amount of intracellular GSSG and the GSH/GSSG ratio did not differ significantly among clones. However, when data were analyzed by the post-test for trend, there was a significant linear reduction (p=0.0023) in the GSH/GSSG ratio in a mutation-dependent manner G12C > G12D > G12V.

Compared to WT, KRAS G12C showed a significant increase of L-alpha-aminobutyric acid (see supplemental Table 1), a key intermediate in the biosynthesis of ophthalmic acid (OPA), indicated as a biomarker of oxidative stress [15, 16]. OPA is an analog of GSH with the thiol group replaced with a methyl group. OPA can be synthesized from 2-aminobutyrate and glutamate by the enzyme c-glutamyl-cysteine-synthetase (GCS) to form c-glutamyl-2 aminobutyrate, which can be catalyzed by glutathione synthetase (GS) to form OPA. To confirm the different activation of the glutathione pathway in the KRAS mutant clones, we validated the intracellular level of OPA in all clones by LC-SRM-MS (Figure 5). There was a significant decrease in OPA levels in KRAS mutant clones towards WT, with a higher level of OPA in the G12C clone than in G12D and G12V.

Since our untargeted metabolomic data suggested that both glutaminolysis and glutathione pathways might be affected, probably through the key role of glutamine, we examined the glutamine dependency of our KRAS clones.

**Figure 6:**
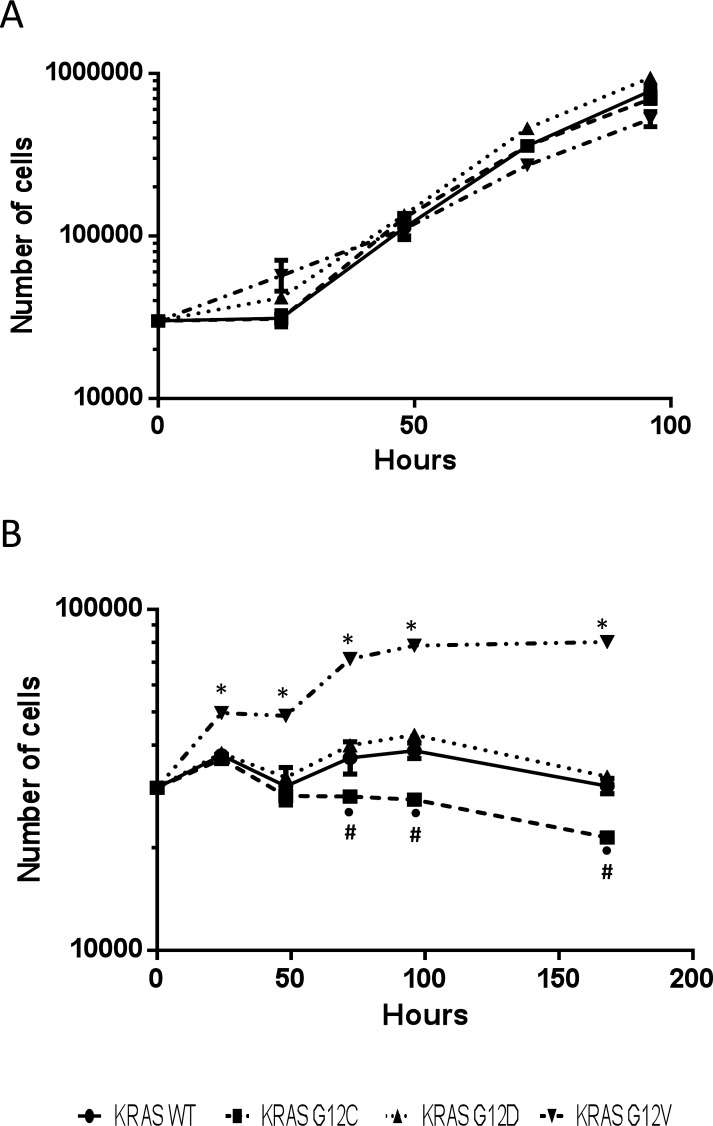
Growth curves of H1299-derived KRAS overexpressing clones cultured with (A) or without (B) glutamine. Cells were cultured in six-well plates and counted 24, 48, 72, 96 and 168 hours after seeding using cell counter. Values are the mean and SD of three independent experiments. Asterisks mark significant differences (two-way ANOVA and Bonferroni post-test, p <0.001), •KRAS G12C vs KRAS WT; *KRAS G12V vs KRAS WT, KRAS G12C, KRAS G12D; #KRAS G12C vs KRAS G12D.

**Figure 7:**
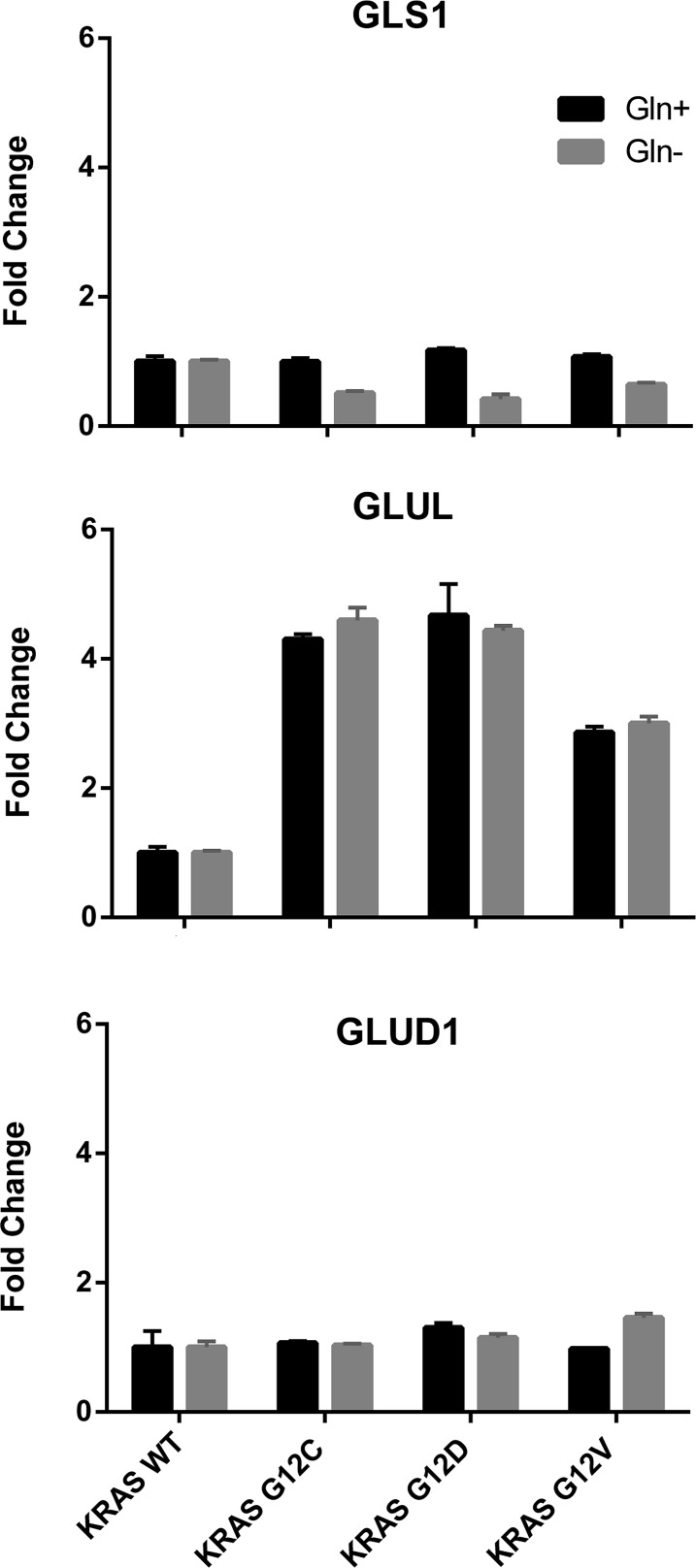
Relative expression levels of genes encoding glutamine-metabolizing enzymes in the indicated clones determined by real-time PCR, with (black bars) or without (grey bars) glutamine. WT clones was arbitrarily set to 1. GLS1, glutaminase; GLUL, glutamine-ammonia ligase; GLUD1, glutamate dehydrogenase.

Results of glutamine deprivation on cell growth in vitro (Figure 6) showed that removal of glutamine strongly reduced the growth of both WT and mutant KRAS expressing clones. However, there were some differences among the clones: the KRAS G12C mutant was more glutamine-dependent than the others, while the G12V clone showed the lowest dependency. To further investigate whether enzymes in the glutaminolysis pathway were responsible for rate-limiting glutamine consumption, we used quantitative rtPCR to analyze the expression of genes encoding key enzymes such as glutamine-ammonia ligase (GLUL), glutaminase (GLS1) and glutamate dehydrogenase (GLUD1). GLUL mediates the capture of an ammonia group by glutamate to synthesize glutamine, while GLS1 catalyzes the breakdown of glutamine to glutamate and ammonia. Then GLUD1 converts glutamate into alpha-ketoglutarate. The expression of GLUL was significantly enhanced in the mutant KRAS clones, while the expression of GLS1 and GLUD1 did not significantly differ among all clones (Figure 7). This expression pattern did not change even when clones were grown without of external glutamine.

## DISCUSSION

Remodeling of metabolism is a recognized hallmark of cell transformation that supports the sustained proliferation of cancer cells and metabolic interventions are now emerging as potential therapeutic target [17]. Oncogene activation plays a role in metabolic reprogramming of cancer cells [18, 19].

KRAS is one of the genes that is often mutated in human cancer, and the mutation is associated with increased malignancy. Its role as a negative and prognostic factor is established in some tumors, while in NSCLC is still debated, perhaps because the majority of clinical studies addressing its specific role were retrospective and mutational status was examined only in a small proportion of patients. We have prospectively demonstrated a negative predictive role of KRAS mutation in first line therapy in NSCLC [7].

To complicate the picture, evidence is emerging that not all the mutations in KRAS have the same biological impact. This, together with the notion that different tumors have different distributions of KRAS mutations, implies that the simple definition of KRAS-mutated tumors may not be sufficient as a marker of response or malignancy. In NSCLC patients, the majority of mutations occurs at codon-12, with a prevalence of G12C which accounts for approximately 40%. At the preclinical level the different KRAS codon-12 mutations induce different responses to some anticancer agents [8]. An important question is whether the different KRAS mutations affect the cell metabolism differently.

To answer to this question, we adopted an unbiased LC-MS metabolomics strategy to obtain a preliminary overall view of changes in cellular metabolites from NSCLC cells expressing either wild-type KRAS or harboring either one of the KRAS mutations G12C, G12D, G12V. We observed a clear distinction in some pathways between WT KRAS-expressing cells and all the other mutants. This is in line with reports about metabolic remodeling driven by tumor cells and KRAS mutations, supporting the feasibility and robustness of our explorative untargeted LC-MS strategy.

The main alterations associated with mutant KRAS involved glycerolphospholipids and amino acids, suggesting deregulated lipogenesis and pronounced energy metabolism characterizing the mutants. Although we did not pursue the lipid species profiling of the clones, the general effect on glycerophospolipid content is in line with the notion that lipogenesis and changes in lipid biochemistry contribute to cancer by multiple mechanisms [20, 21].

The impact of Ras on lipid metabolism has emerged only recently. Ras-transformed cells rely heavily on fatty acid uptake and utilization of lysophospholipids, that renders Ras-driven cancer cells particularly prone to maintaining their growth [22]. The pronounced changes in amino acid levels we observed is again in accordance with cancer cell metabolic reprogramming, which is manifested as altered nutrient uptake and use. Glycolysis and glutaminolysis are the two central pathways that fuel cancer metabolism. Although the Ras oncogene promotes glycolysis in many cells and tumor contexts [9, 13, 23], our metabolomic results and extracellular lactate levels gave no clue to significantly heightened glycolysis in these KRAS mutants (supplemental Figure 4).

Glutamine serves as a major anaplerotic substrate for the TCA cycle and KRAS-transformed cells are closely dependent on glutamine for growth and survival [24]. In accordance with this, we found that KRAS mutants had low intracellular levels of glutamine and glutamate, suggesting sustained biosynthetic reactions and a prominent role of glutaminolysis. However, the mutants’ dependence on glutamine was different. The KRAS G12C clone was most susceptible to glutamine deprivation, and KRAS G12V was less. This different dependence on exogenous glutamine was not explained by differences in transcriptional levels of enzymes in glutaminolysis, although these mutants had comparably enhanced expression of GLUL transcript. This specific genetic make-up suggests a special ability to fix free nitrogen in the form of ammonia by converting glutamate to glutamine, counteracting glutamine deprivation [25]. Consistent with this pathway, the KRAS mutants showed a lowered intracellular asparagine that might call for the nitrogen anabolism underlying glutaminolysis in these clones.

Glutaminolysis also supports the production of molecules such as glutathione which protect cells from oxidative stress. The intracellular redox state of the clones was therefore examined investigating the ratio of reduced to oxidized glutathione (GSH/GSSG). KRAS mutant clones had a redox state comparable to the WT and small, but significant, differences were mainly observed among mutations at codon-12. Altered detoxification status in KRAS-transformed fibroblasts has been reported, with better detoxification potential in G12D and G12V mutants than their control [26].

We characterized the antioxidant profile of our clones by measuring ophthalmate (OPA) which is an endogenous analog of GSH, indicated as a potential biomarker for GSH depletion [15]. The G12C clone had a high level of OPA compared to G12D and G12V mutants (but similar to the WT cells), suggesting that G12C may rely on a strong GSH/OPA redox buffer system. This suggests that OPA can act as GSH analog for functions that do not require the thiol group (as in GSH). This would provide a functional meaning for its synthesis during oxidative stress rather than simply a consequence of GSH depletion [16].

Overall our data further support the notion that KRAS mutational status involves a general metabolic reprogramming to fuel growth and counter stress, including enhanced amino acids catabolism, alterations in lipid biochemistry and antioxidant program.

Our evidence was obtained using a robust isogenic system generated by stable transfection of WT or mutated KRAS. Although the system has some limitations, as it was generated by overexpressing KRAS (WT or mutated) in a WT KRAS-expressing cell line, it has several advantages over cell lines with different KRAS status. In fact it allows us to determine, in a similar genetic background, the role of a single point mutation in KRAS. The system was generated to express similar levels of wild-type or mutant protein and relies on the evidence that two independent clones, generated for each mutation, have similar growth rates, protein expression and GTPase activity, thus reducing the risks of clonal selection possible with stable transfectants.

The particular metabolic adaptations of KRAS mutational status might contribute to their different responses to anticancer treatments [27] and might be exploited as novel metabolic vulnerabilities for potential treatment modalities.

## METHODS

### Cell cultures and growth curve experiments

Human non-small-cell lung carcinoma NCI-H1299 KRAS overexpressing clones were grown in RPMI1640 medium with 500 microG/mL of G418 (Gibco) added. Cells were maintained at 37°C in a humidified atmosphere of 5% (v/v) CO_2_ in air. For growth curve experiments, cells were cultured in six-well plates with or without glutamine and were counted 24, 48, 72, 96 and 168 hours after seeding, using a cell counter (Beckman). The growth curves were plotted as absolute numbers of cells. Each experiment consisted of three replicates for each point and the plotted data are the mean and SD of three independent experiments. Statistical analyses (two-way ANOVA and Bonferroni post-test for multiple comparison) were done using GraphPad Prism software (GraphPad Prism, v.6.01).

### KRAS expression level and activation

KRAS expression level in all clones was evaluated by Western blotting analysis. Briefly, cells were plated at different concentrations in 100-mm tissue culture dishes (Sterilin). Forty-eight hours after seeding, extracts were prepared by lysing cells for 30 minutes on ice in protein lysis buffer in the presence of protease inhibitors. Insoluble material was pelleted at 13,000x*g* for 10 min at 4°C and the protein concentration was determined using a BioRad assay kit (BioRad). A 50-microG sample of total cellular proteins was separated on SDS-PAGE and electrotransferred to PVDF membrane (Millipore). Antibodies were diluted following the manufacturer’s instructions in 5% non-fat dry milk in TBS-Tween 20 0.05% (TBS-T). Immunoblotting was carried out with the following antibodies: anti-KRAS and anti-actin, obtained from Santa Cruz Biotechnology. Antibody binding was detected using peroxidase secondary conjugated antibodies (Santa Cruz Biotechnology) and visualized by enhanced chemiluminescence (ECL, Amersham).

Active (GTP-bound) KRAS was measured in mutated and WT expressing cells by the KRAS Activation assay Kit (Cell Biolabs) according to the manufacturer’s instructions. 1000 microG of whole-cell cleared lysate was incubated with RAS-RAF binding domain for 60 min at 4°C. The complexes were collected by centrifugation and washed. Proteins were separated by SDS-PAGE, followed by Western blot. The KRAS protein was detected with anti-KRAS antibody (Santa Cruz Biotechnology). Actin was detected as a loading control.

### Metabolomic sample preparation

For metabolomics analysis, NCI-H1299 KRAS overexpressing clones were grown for 48 hours in biological triplicate. At 48 hours, all four clones have the same proliferation rate. Metabolites were extracted from clones as reported [28], with minor modifications. Briefly, NSCLC cells, of each clone (three biological replicates/clone), were rapidly rinsed in saline solution (~ 2s), aspirated, and metabolism was quenched by adding ~15mL of liquid N_2_ to the dish. The plates were then stored at −80°C, and extracted and analyzed within seven days. Extraction was done by adding 1 mL of ice-cold 90% 9:1 MeOH:CHCl_3_ to each plate and cells were scraped. The extraction solvent contained tryptophan-D8, 3-hydroxyindoleacetic acid-D5, methionine-D3, 17-alpha-methyltestosterone-D3, fludrocortisone and desoximetasone as internal standards (1 microM/each) to ensure metabolite extraction, injection and chromatographic consistency for positive and negative ionization modes. Extracts were transferred to 1.5 mL micro-centrifuge tubes and pelleted at 4°C for 15 min at 10000xg. Supernatants were divided into two aliquots, dried, then reconstituted in acidic or basic LC-compatible solvent.

### Metabolomic profiling by LC-MS

A portion (2 microL) of metabolite extract from all the KRAS clones was directly analyzed by LC-MS/MS, using an LTQ Orbitrap XL™ (Thermo Scientific), interfaced with a 1200 series capillary pump (Agilent). The MS instrument was operated in positive (POS) and negative (NEG) ionization modes. Chromatographic and MS conditions were as reported [29]. Untargeted metabolomic data were processed using the MS label free differential analysis software SIEVE v1.3 (ThermoFisher). SIEVE was run on all the LC–MS full-scan chromatograms using the component extraction setting. The chromatograms were time-aligned, blank subtracting (solvent background) and referencing the sample acquired in the middle of the sequence. The framing parameters were set at 0.01 Da for the m/z window and 0.35 min for the retention time (RT) window; 500,000 was used as the intensity threshold. Before any statistical analysis the value of each molecular species (frame) detected by SIEVE was normalized to the intensity of the internal standards using the FRAME option for spiked internal standards (all frames are normalized to the designated frame with the internal standard ion). An additional filtering criterion was then applied to include in the dataset only frames with an intensity coefficient of variation <50% in at least one experimental group.

### Multivariate metabolomic data analysis

The normalized ion intensity data for each clone were submitted to the SIMCA-P13 software package (Umetrics) for multivariate data analysis. The variables were scaled using Pareto scaling to increase the low abundance ions without significantly amplifying the noise. To maximize class discrimination, the data were analyzed by orthogonal partial least-squares discriminant analysis (OPLS-DA). S-plots were calculated to visualize the relationship between covariance and correlation within the OPLS-DA results. Variables that significantly contributed to discrimination between groups were identified.

### Identification of metabolites

For metabolite identification, the frame m/z values were used for batch searches on the METLIN database (http://metlin.scrpss.edu) and Human Metabolome Database (HMDB, http://www.hmdb.ca/). Accurate mass data and isotopic distribution for the precursor and product ion were compared to spectral data of the reference compounds in the databases. Lipids were tentatively identified by high mass accuracy and MS/MS fragment ions using the LIPID Mass database without authentic standards. Identifications were reported only for metabolites with accurate mass match < 5 ppm.

### Mapping metabolic pathways

For biological interpretation of the metabolite dataset by our untargeted strategy, we mapped the identified metabolites to the KEGG pathway database (Kyoto Encyclopedia of Genes and Genomes; (www.genome.jp/kegg/), using MetaboAnalyst 2.0, a comprehensive online tool suite for metabolomic data analysis and interpretation (www.metaboanalyst.ca).

### Measurement of intracellular oxidized and reduced glutathione and ophthalmic acid by LC-MRM-MS

Glutathione (GSH, GSSG) and OPA were quantified by LC-MRM-MS [30, 31], with minor modifications as detailed in Supplemental Methods. Statistical analysis was done using one-way ANOVA and Tukey-Kramer post-hoc test (GraphPad Prism software).

### Extracellular lactate

One-hundred microL of conditioned cell culture media from KRAS-overexpressing clones were filtered through a 10 kDa MW spin filter (Millipore) to remove proteins and used to determine lactate secretion. Lactate was measured using the Lactate Colorimetric Assay Kit (Abcam). Statistical analysis (one-way Anova) was done using GraphPad Prism software.

### Real-time PCR

RNA from H1299 KRAS-overexpressing clones grown for 48 hours with or without glutamine was purified using the Simply RNA Maxwell Total RNA Purification Kit (Promega). Retrotranscription to cDNA was done using the High Capacity cDNA Retrotranscription Kit (Applied Biosystems). Optimal primer pairs for selected genes (supplemental Table 2), spanning splice junctions, were chosen using PRIMER-3 software (http://bioinfo.ut.ee/primer3-0.4.0/primer3/input.htm) and the specificity was verified by detecting single-band amplicons of the PCR products. cDNA was amplified by real time RT-PCR (ABI-7900, Applied Biosystems) with the SYBR Green technique. Relative quantification of mRNA was done using the ΔΔCt method. Actin was used as reference gene and H1299 KRAS WT clone was arbitrarily set to 1.

## SUPPLEMENTARY METHODS, MATERIAL, FIGURES AND TABLES





## ABBREVIATIONS

GLS1: glutaminase
GLUD1: glutamate dehydrogenase
GLUL: glutamine-ammonia ligase
GSH: reduced glutathione
GSSG: oxidized glutathione
KRAS G12C: NSCLC cells overexpressing the mutant form of KRAS at codon-12 (G12C)
KRAS G12D: NSCLC cells overexpressing the mutant form of KRAS at codon-12 (G12D)
KRAS G12V: NSCLC cells overexpressing the mutant form of KRAS at codon-12 (G12V)
KRAS WT: NSCLC cells overexpressing the wild-type form of KRAS
LC-MS: Liquid-Chromatography Mass Spectrometry
LC-MRM-MS: Liquid-Chromatography Multiple Reaction Monitoring coupled with Mass Spectrometry
NSCLC: Non-small-cell-lung cancer
OPA: ophthalmic acid
OPLS-DA: orthogonal partial least-squares discriminant analysis
PC: phosphatidylcholines
PI: phosphatidylinositols
PS: phosphatidylserine
TCA: tricarboxylic acid.
