# Supplementary material for: Capturing the metabolomic diversity of KRAS mutants in non-small-cell lung cancer cells
Source: Oncotarget. 2014 May 12;5(13):4722–31. doi: 10.18632/oncotarget.1958 (PMC4148094; doi:10.18632/oncotarget.1958)
Supplement: Supplementary file 1 [file oncotarget-05-4722-s001.pdf]

Capturing the metabolomic diversity of KRAS mutants in non-small-cell lung cancer cells

SUPPLEMENTAL DATA

METHODS

Measurement of intracellular reduced glutathione (GSH), oxidized glutathione (GSSG) and ophthalmic acid (OPA) by Liquid Chromatography-Multiple Reaction Monitoring-Mass Spectrometry (LC-MRM-MS).

NCI-H1299 KRAS overexpressing clones were grown for 48 hours in biological triplicate. Cells were rapidly rinsed in saline solution (~ 2s), aspirated, and metabolism was quenched by adding ~15 mL of liquid N_2 to the dish. The plates were stored at -80°C, and extracted and analyzed within seven days. Metabolites were extracted by adding 1 mL of cold 10% TCA to cell plates and the cells were scraped. All the steps were conducted in ice, to minimize GSH oxidation or enzymatic degradation. Extracts were centrifuged at 10000xg for 10 min at 4°C, the supernatants were collected and filtered on a syringe filter unit (0.22 mm). OPA were extracted from each NSCLC clone by adding 1 mL of cold (-20°C) methanol to each cell dish, the cells were scraped, and extracts were collected and centrifuged at 10000xg for 10 min. The supernatants were removed and dried then reconstituted in 0.1% formic acid. All supernatants were analyzed directly by LC–MS/MS with the Agilent 1200 series system for LC. Separation was with an Ascentist Express Peptide ES-C18 column (Supelco Analytical; 10 cmx2.1 mm, 2.7 microm) using 0.1 % formic acid in water as mobile phase A and 100% acetonitrile as mobile phase B at a flow rate of 0.2 mL/min. Elution started with 99% of A and 1% of B, followed by a 16-min linear gradient to 99% of B, 2-min isocratic elution and a 1-min linear gradient to99 % of A, which was maintained for 8 min to equilibrate the column.
Mass spectrometric analysis was done using an Agilent 6410 triple quadrupole mass spectrometer (Agilent Technologies) in positive ion mode for GSH, GSSG and OPA. MRM for GSH, GSSG and OPA was done with collision energy optimized for each transition as reported in the table below. Quantitative analyses were processed with MassHunter workstation quantitative analysis software vB.01.04 (Agilent Technologies).

Table. Chromatographic retention times and optimum MS source and analyzer conditions for MRM analysis of GSH, GSSG and OPA. GSH, oxidized glutathione; GSSG, reduced glutathione, OPA, ophthalmic acid.

Chemicals	RT (min)	Source voltages (V)		Precursor ion *(m/z)*	Product ion I *(m/z)* and collision energy (eV)	Product ion II *(m/z)* and collision energy (eV)
		Fragmentor	*Dwell*		*(Quantification ion)*	*(Confirmation ion)*
GSH	2.1	80	80	308	162 (10)	179 (10)
GSSG	3.6	80	80	307	130 (15)	231 (15)
OPA	2.2	80	80	290.1	130 (20)	227 (12)

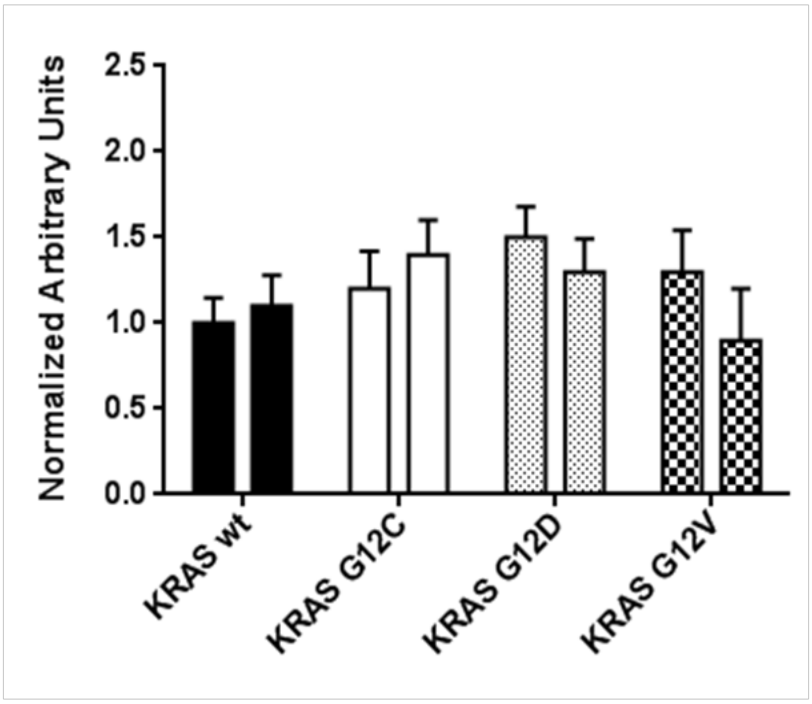

Supplemental Figure 1: KRAS protein expression in NCI-H1299-derived isogenic clones analyzed by Western blot. Protein bands were quantified by scanning densitometry. Expression data were normalized to actin. Each bar represents the normalized volume density (mean ± SD, 3 independent experiments). The vertical axis shows arbitrary density units.

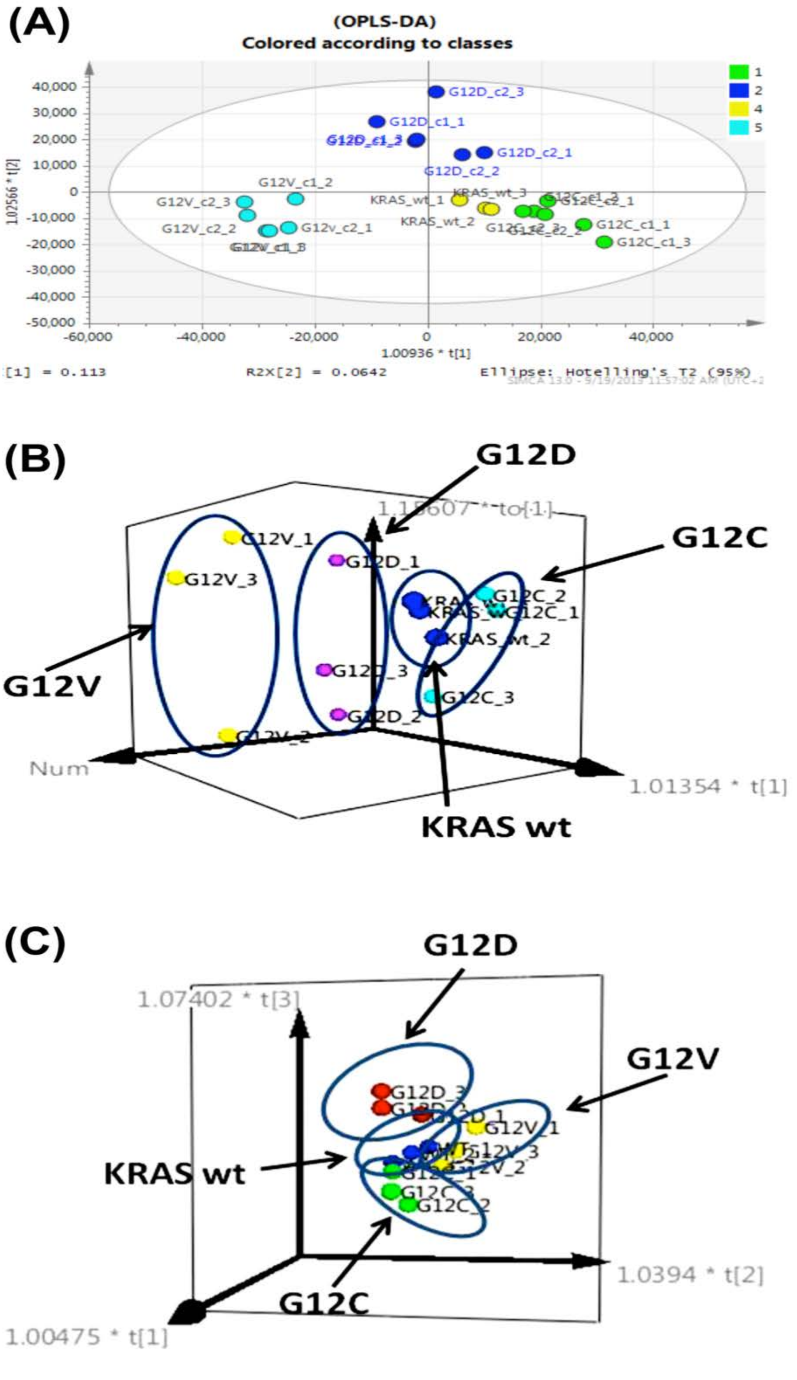

Supplemental Figure 2: Multivariate analysis of metabolomic profiles from over-expressing KRAS mutant clones (G12C, G12D, G12V) and WT. Panel A). OPLS-DA score plot showing classes separed according to their metabolic species signature, where classes correspond to two different clones for each KRAS mutational status (c1, clone 1; c2, clone2). Panels B) and C). 3D OPLS-DA score plots showing metabolomics signatures clustered according to KRAS mutational status (only one clone for each mutant) for positive and negative ionization mode.

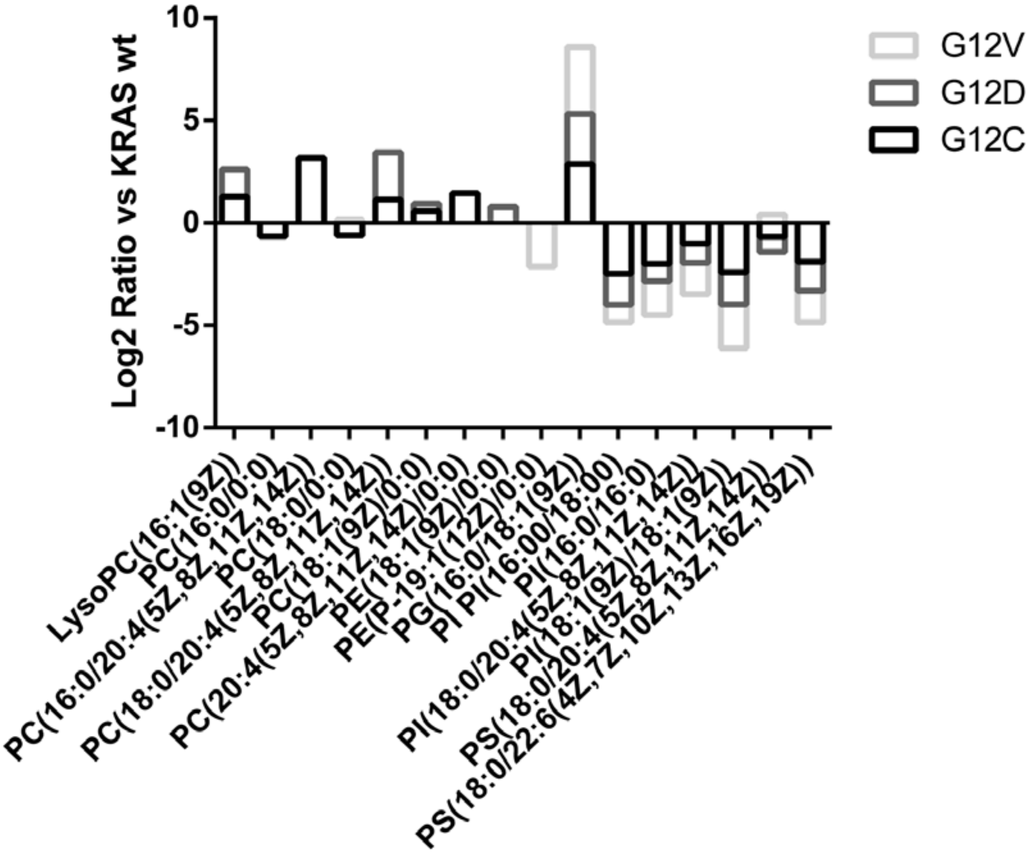

Supplemental Figure 3: Glycerophospholipid distribution expressed as the ratio fold-change (log2 ratio) in overexpressing KRAS mutant clones (G12C, G12D, G12V) vs WT. G12C black bar, G12D red bar, G12V green bar. Glycerophospolipids were tentatively identified only using the LipidMaps database (http://www.lipidmaps.org).

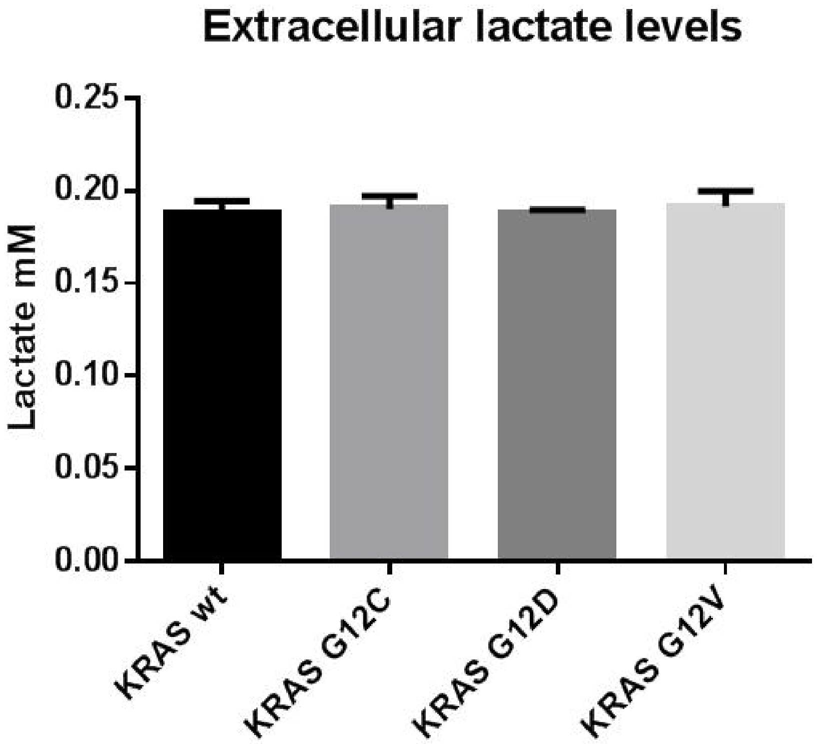

Suppemental Figure 4: Lactate levels in the medium of KRAS-overexpressing clones grown for 48 hours in regular media. Lactate was measured using the Lactate Colorimetric Assay Kit (Abcam). Data are expressed as milliM mean ± SD (3 biological replicates). Data were analyzed with one-way ANOVA (GraphPad Prism, v.6.01).
